# Will AI Replace Physicians in the Near Future? AI Adoption Barriers in Medicine

**DOI:** 10.3390/diagnostics16030396

**Published:** 2026-01-26

**Authors:** Rafał Obuchowicz, Adam Piórkowski, Karolina Nurzyńska, Barbara Obuchowicz, Michał Strzelecki, Marzena Bielecka

**Affiliations:** 1 Department of Diagnostic Imaging, Jagiellonian University Medical College, 30-663 Krakow, Poland; 2 Department of Biocybernetics and Biomedical Engineering, AGH University of Krakow, 30-059 Krakow, Poland; pioro@agh.edu.pl; 3 Department of Algorithmics and Software, Silesian University of Technology, 44-100 Gliwice, Poland; karolina.nurzynska@polsl.pl; 4 Department of Conservative Dentistry with Endodontics, Jagiellonian University Medical College, 31-155 Krakow, Poland; barbara.obuchowicz@uj.edu.pl; 5 Institute of Electronics, Lodz University of Technology, 93-590 Lodz, Poland; michal.strzelecki@p.lodz.pl; 6 Department of Algorithmics, Faculty of Geology, Geophysics and Environmental Protection, AGH University of Krakow, 30-059 Krakow, Poland; bielecka@agh.edu.pl

**Keywords:** artificial intelligence, medicine, radiology, large language models, physical examination, out of distribution generalization, legal liability, clinical augmentation

## Abstract

**Objectives**: This study aims to evaluate whether contemporary artificial intelligence (AI), including convolutional neural networks (CNNs) for medical imaging and large language models (LLMs) for language processing, could replace physicians in the near future and to identify the principal clinical, technical, and regulatory barriers. **Methods**: A narrative review is conducted on the scientific literature addressing AI performance and reproducibility in medical imaging, LLM competence in medical knowledge assessment and patient communication, limitations in out-of-distribution generalization, absence of physical examination and sensory inputs, and current regulatory and legal frameworks, particularly within the European Union. **Results**: AI systems demonstrate high accuracy and reproducibility in narrowly defined tasks, such as image interpretation, lesion measurement, triage, documentation support, and written communication. These capabilities reduce interobserver variability and support workflow efficiency. However, major obstacles to physician replacement persist, including limited generalization beyond training distributions, inability to perform physical examination or procedural tasks, susceptibility of LLMs to hallucinations and overconfidence, unresolved issues of legal liability at higher levels of autonomy, and the continued requirement for clinician oversight. **Conclusions**: In the foreseeable future, AI will augment rather than replace physicians. The most realistic trajectory involves automation of well-defined tasks under human supervision, while clinical integration, physical examination, procedural performance, ethical judgment, and accountability remain physician-dependent. Future adoption should prioritize robust clinical validation, uncertainty management, escalation pathways to clinicians, and clear regulatory and legal frameworks.

## 1. Introduction

Rapid advances in artificial intelligence (AI) have transformed what is technically possible in medicine, prompting claims that algorithms could eventually replace clinicians in selected domains. Yet, the evidence base remains fragmented across specialties, often limited to narrow tasks, single-center datasets, and retrospective designs, with uncertain generalizability to real-world care. In parallel, legal liability, evolving regulation, data governance, and workflow integration pose unresolved questions in high-stakes clinical environments. The striking achievements of deep learning in medical imaging and the communication capabilities of large language models coexist with well-documented limitations—out-of-distribution failures, opacity, hallucinations, and the absence of embodied examination and sensory inputs essential to clinical reasoning. High-profile deployment setbacks further underscore the gap between research performance and safe, scalable implementation.

This work is warranted to provide a balanced, interdisciplinary appraisal of whether—and to what extent—AI can substitute for, or should instead augment, human clinicians. We synthesize technical, clinical, ethical legal, and organizational evidence; identify failure modes and implementation barriers across multiple fields; and propose a pragmatic framework for evaluating clinical utility, safety, and cost effectiveness.

In this review, the term “near future” is used to denote an approximate time horizon of 5 to 10 years, corresponding to a typical technological and regulatory cycle in medicine. This period reflects a realistic interval within which current AI architectures—such as convolutional neural networks and large language models—could mature, be validated in clinical trials, and undergo regulatory approval for broader clinical adoption. Developments extending beyond this range (10–20 years) are considered speculative and lie outside the foreseeable scope of this analysis.

In clinical practice, AI systems are incorporated into imaging workflows as assistive diagnostic tools rather than autonomous decision-makers. It should be emphasized that these deployments are limited to narrowly defined, well-controlled tasks such as triage, prioritization, or decision support and do not constitute autonomous clinical decision-making. In all reported implementations, the physician remains responsible for integrating algorithmic outputs with clinical context and for issuing the final diagnostic or therapeutic decision. Typically integrated within the hospital PACS (Picture Archiving and Communication Systems) or radiology information system (RIS), they function as background algorithms that automatically process incoming examinations to identify abnormal findings or prioritize potentially urgent cases for immediate review. In radiology, for example, AI-based triage systems can flag examinations showing signs of intracranial hemorrhage, pulmonary embolism, or pneumothorax, ensuring that critical studies are displayed earlier in the radiologist’s worklist. FDA-cleared algorithms such as Aidoc and Viz.ai have demonstrated significant improvements in workflow efficiency and diagnostic timeliness for pulmonary embolism and stroke detection in CT (Computed Tomography) imaging, reducing time-to-notification by up to 40% [1,2]. Likewise, deep learning systems for mammography screening—for instance, Transpara (Screen Point Medical) and Lunit INSIGHT MMG—have shown comparable or superior performance to radiologists in detecting breast cancers, supporting earlier recall and reducing false negatives [3,4]. The alert mechanisms are graphical in nature—most often visual depictions such as color overlays, bounding boxes, or probability heatmaps are superimposed onto the image to indicate regions of algorithmic concern. These annotations are designed to assist, not replace, the radiologist’s judgment. The physician reviews the AI suggestions directly within the diagnostic console, confirms or rejects them based on full clinical context, and ultimately issues the verified report. In all cases, the final diagnostic decision and responsibility for patient management remain with the clinician. This workflow model illustrates AI’s role as a supportive collaborator that enhances efficiency and vigilance while preserving human oversight. By pre-analyzing large imaging datasets, automating measurements, and highlighting atypical patterns, AI systems help radiologists maintain accuracy under high workloads [5]. As a result, clinicians experience measurable reductions in both cognitive fatigue and physical strain, enabling greater consistency, faster turnaround times, and improved allocation of professional attention to complex cases requiring nuanced human interpretation. As a result, clinicians may experience reduced cognitive load and improved workflow efficiency, primarily due to automated pre-analysis, measurement assistance, and prioritization of urgent cases rather than a direct reduction in diagnostic responsibility.

While this review devotes substantial attention to imaging-based artificial intelligence, this emphasis reflects the current state of clinical deployment rather than an underestimation of large language models (LLMs). Convolutional neural network-based systems in radiology and pathology represent the most mature, regulated, and empirically validated AI applications in medicine, with routine integration into clinical workflows. In contrast, LLMs, despite impressive performance in medical examinations, documentation, and patient-facing communication, remain primarily supportive tools whose clinical impact is constrained by hallucinations, lack of grounding in physical examination, and unresolved accountability. Accordingly, the manuscript emphasizes imaging as the most advanced domain of medical AI, while using LLMs as a complementary case study illustrating both the promise and the limitations of language-based systems in clinical practice.

To sharpen the scope of this analysis, this review does not ask whether artificial intelligence can replace physicians in general, but under what restricted conditions AI systems may approximate physician-level performance. Based on current evidence, such approximation is plausible only in narrow, well-defined clinical tasks characterized by standardized inputs, large and representative training datasets, stable operating conditions, and objectively measurable endpoints—such as image-based detection, segmentation, or triage in radiology and pathology. In contrast, tasks requiring physical examination, multimodal sensory integration, procedural skills, contextual judgment, or legal and ethical accountability remain fundamentally human-dependent. By explicitly distinguishing between domains where AI can achieve near-human technical performance and those where structural clinical constraints persist, this review frames physician replacement not as a binary outcome, but as a task- and context-dependent question. Representative clinical applications of artificial intelligence and their implications for physician replacement are summarized in Table 1.

By distinguishing where AI adds reliable value from where human expertise remains indispensable, the analysis aims to guide clinicians, developers, and policymakers toward responsible adoption that enhances patient outcomes without eroding trust or the patient–physician relationship.

A central distinction underlying this review is that between task-level automation and physician-level replacement, as summarized in Table 2. Task-level automation encompasses narrowly defined, standardized activities—such as image triage, lesion detection, quantitative measurement, or documentation drafting—that can be reliably delegated to AI systems operating under controlled conditions and human oversight. In contrast, physician-level replacement would require autonomous performance across the full scope of clinical practice, including integration of heterogeneous data, physical examination, contextual reasoning, procedural skills, ethical judgment, and legal accountability. While current AI systems demonstrate near-human or superior performance in selected task categories (Table 1), they remain structurally incapable of assuming physician-level roles. Accordingly, contemporary AI enables scalable task automation and clinical augmentation, rather than replacement of physicians as accountable clinical decision-makers.

## 2. Methods

This study was conducted as a narrative review of the scientific literature examining the potential for artificial intelligence (AI) to replace or augment physicians in clinical medicine. A structured literature search was performed in PubMed/MEDLINE, Web of Science, Scopus, IEEE Xplore, and Google Scholar, covering publications from January 2015 to September 2025. Search terms combined AI methodologies (e.g., artificial intelligence, deep learning, convolutional neural networks, and large language models) with clinical application domains (e.g., radiology, medical imaging, and clinical decision support) and implementation-related concepts (e.g., generalization, out-of-distribution performance, human-in-the-loop, legal liability, and regulation).

Peer-reviewed original studies, narrative and systematic reviews, meta-analyses, and authoritative position papers were included if they addressed AI performance, clinical validation, generalizability, ethical or legal constraints, or workflow integration in healthcare. Reference lists of key articles were manually screened to identify additional relevant studies. Evidence was synthesized thematically rather than quantitatively, reflecting the interdisciplinary and conceptual scope of the review.

Given the narrative design and the aim to integrate technical, clinical, ethical, and legal perspectives, formal systematic review procedures and PRISMA flow diagrams were not applied. Discrepant or conflicting findings were explored and discussed qualitatively to highlight contextual dependencies and limitations, rather than being excluded from the analysis.

### 2.1. Eligibility Criteria

Studies were included if they met at least one of the following criteria: empirical clinical validation of artificial intelligence (AI) systems (e.g., diagnostic accuracy, workflow impact, or reproducibility); comparative evaluations between AI systems and human clinicians; investigations addressing model generalization, robustness, bias, or out-of-distribution performance; studies assessing large language model performance in medical examinations, patient communication, or clinical documentation; and analyses of legal, ethical, regulatory, or liability frameworks for medical AI, particularly within European Union and U.S. FDA contexts. High-quality systematic reviews, meta-analyses, consensus statements, and authoritative position papers by leading AI or medical researchers relevant to physician replacement or clinical augmentation were also included. Excluded were non-peer-reviewed opinion pieces lacking scholarly grounding, AI studies unrelated to healthcare or clinical decision-making, purely technical AI papers without direct clinical relevance, studies limited to animal models or non-clinical simulations, and marketing materials without independent validation.

### 2.2. Study Selection and Synthesis

Titles and abstracts were screened for relevance, and full-text articles were reviewed when abstracts met the inclusion criteria. The final corpus of studies was organized thematically rather than statistically pooled, reflecting the narrative and interdisciplinary scope of the review. Evidence was synthesized across key domains, including diagnostic performance and reproducibility, model generalization and failure modes, human sensory and embodied limitations of artificial intelligence, legal and regulatory constraints, workflow integration and clinician oversight, and ethical and patient-centered considerations. Discrepant or conflicting findings were explored and discussed qualitatively to highlight contextual dependencies and limitations rather than being excluded from the analysis. Given the narrative review design, PRISMA flow diagrams were not applied; however, methodological transparency was ensured through explicit reporting of databases, time frames, search terms, and inclusion logic, in accordance with MDPI editorial guidance for narrative reviews.

### 2.3. Data Source and Their Presentation

Throughout this review, reported performance metrics such as AUC, sensitivity, specificity, and F1-score are drawn from heterogeneous studies that differ substantially in dataset composition, patient populations, imaging protocols, reference standards, and evaluation methodologies. These values are therefore presented to illustrate typical performance ranges within narrowly defined tasks rather than to enable direct quantitative comparison across algorithms or clinical domains. Typical performance ranges reported across major imaging modalities are summarized in Table 3, illustrating both the strengths of AI in narrowly defined tasks and the heterogeneity of evaluation settings. High numerical performance in isolated studies should not be interpreted as evidence of general clinical equivalence or readiness for autonomous deployment.

## 3. Phenomena Supporting the Potential Replacement of Humans by AI in Medicine

Speculation about replacing physicians with artificial intelligence is fueled by the capabilities of deep learning systems based on convolutional neural networks and Transformer-based architectures. It should be emphasized that several AI tools—such as Aidoc for pulmonary embolism triage and Lunit INSIGHT for mammography or Satori Platform (Test Dynamics) for MRI, CT and CR—which are already routinely deployed in clinical radiology workflows across multiple institutions. Their adoption demonstrates that AI has moved beyond experimental research and into everyday clinical practice in selected, well-defined domains.

Convolutional neural networks (CNNs), particularly when combined with advanced decision-tree architectures, enable highly accurate detection of image patterns, understood as groups of shapes and textures. This capability underpins their success in radiology [6,7]. Multiple studies have shown that such networks can identify pathological tissue changes in a highly repeatable manner and, in some tasks, with greater precision than human observers. One contributing factor is interobserver variability: even experienced radiologists may interpret the same imaging finding differently [8,9]. When trained using supervised metric learning approaches, CNN-based systems can assess lesions more reproducibly and consistently, with reduced susceptibility to subjective interpretation. This highlights a relative limitation of human observers and provides a strong argument for the use of automated systems in lesion detection and quantitative assessment [10].

Another feature that strongly appeals to proponents of artificial intelligence is the associative and compressive capacity of these models, namely their ability to detect similarities and latent patterns within images. Importantly, AI systems can identify features that are not apparent even to trained radiologists. Despite the ability to analyze features of medical images only—understood as intensities and affinities of bitmap pixels (and lacking clinical context), they typically yield pattern-based diagnostic outputs, which are sometimes spectacular, as they can be impossible for physicians to establish themselves.

For example, studies of algorithms applied to chest radiographs have shown that AI can detect osseous lesions or inflammatory lung changes and can also infer metabolic states, as well as patient age and sex, from image information not intended for such analyses [11,12,13]. In a similar manner, demographic attributes—including patient race—can be inferred from chest X-rays, despite the fact that these images are primarily acquired to assess lung parenchyma [14,15].

Comparable findings have been reported in analyses of retinal fundus photographs. AI models have demonstrated the ability to estimate age and sex, identify tobacco use or hypertension, and even approximate HbA1c levels or blood hemoglobin concentration from retinal images [16,17]. These subvisual features, which are difficult or impossible for humans to perceive directly, can nevertheless be captured by machine learning models as clinically relevant information. Their detection has the potential to reduce the need for additional diagnostic tests, limit patient exposure, and lower healthcare costs [18]. Notably, in most of these studies, the systems were not explicitly trained to recognize such attributes. Instead, the extraction of hidden information emerged as a byproduct of networks trained for other primary tasks. These observations are often interpreted as evidence of an apparent “reasoning” capability, whereby AI systems autonomously exploit latent image information to enhance task performance.

This phenomenon should not be interpreted as causal or symbolic reasoning, but rather as the emergence of latent statistical representations learned from large datasets. Such representations may capture clinically relevant correlations, yet they can also reflect confounding factors or dataset-specific artifacts, underscoring the need for careful validation and interpretability analysis.

Among further arguments for applying machine assistance in medicine is Transformer, which has supplanted recurrent networks in natural language processing transformer-based architectures. Transformer-based architectures (e.g., BERT—Bidirectional Encoder Representation from Transformer; GPT—Generative Pretrained Transformer) are typically trained in a self-supervised paradigm—predicting masked portions of text or the next token (a numerical representation of a word or sub-word)—and they constitute the foundation of contemporary large language models (LLMs) [19]. The imagination of AI proponents in medicine is further stimulated by the near perfect performance of language systems on medical examinations. As an example, in USMLE exams, LLMs have surpassed human-level performance (GPT-4 achieved ~90% accuracy on USMLE-style medical questions, far exceeding the typical pass threshold) [20]. High scores on these complex multiple choice tasks indicate advanced abilities to associate information and to handle text proficiently. Performance on standardized examinations primarily reflects pattern recognition and knowledge retrieval within constrained textual formats, rather than real-world clinical reasoning or decision-making under uncertainty. Moreover, such results may be influenced by overlap between training data and publicly available examination preparation materials. The use of LLM’s to enhance learning, including in medical education (aid in repetition and memorizing tasks) is also noteworthy [21]. In contexts where contact with patients is permitted, computer systems—once trained on the diagnoses—are able to provide medical advice that, in patients’ judgment, was more detailed than that offered by physicians. According to observers, the computer systems responded with greater empathy and patience toward patients, offering precise, multi-context explanations delivered in a more accessible manner—tailored to the patient’s level and knowledge [22]. These observations are based largely on evaluations of written responses in controlled or asynchronous settings and do not involve physical examination, responsibility for outcomes, or longitudinal patient care. Perceived empathy in generated text should therefore not be equated with clinical empathy as practiced in real patient–physician interactions. However the capabilities of large language models in analyzing medical data and communicating effectively with patients are frequently cited as the chief arguments supporting the potential replacement of humans in medicine [23].

Overall, the ability of deep learning models to encode clinically relevant latent features supports their role as hypothesis-generating tools in precision medicine but also patient support and training aid in medical schools. However, these same findings emphasize the need for rigorous interpretability and validation to distinguish true pathophysiological signals from dataset-specific correlations.

### 3.1. Empirical Evidence That AI Models Encode Latent, Clinically Relevant Features Beyond Human Annotations

Recent empirical work has shown that deep neural networks trained for narrow imaging tasks frequently learn latent representations that encode clinically meaningful information never explicitly labeled during training.

For instance, models trained solely to detect pneumonia or pleural effusion on chest radiographs have been found to infer patient sex, age, race, and even smoking status—features that radiologists cannot reliably discern [24]. These findings suggest that AI systems capture subtle texture, shape, or intensity patterns correlated with systemic or demographic attributes, raising both opportunities and ethical concerns for fairness and generalization.

Similarly, convolutional networks trained for retinopathy screening or ocular disease classification unexpectedly predict hemoglobin A1c, blood pressure, cardiovascular risk, and smoking history directly from retinal photographs [25]. This capability illustrates that the model’s learned representation encodes vascular and metabolic signatures invisible to clinicians but relevant to systemic health.

In oncology, histopathology networks trained to classify tumor subtypes or grade have demonstrated the ability to infer microsatellite instability, molecular mutations (e.g., IDH1 in gliomas), and patient prognosis without genetic inputs—showing that morphological surrogates of molecular biology are embedded in image textures [26,27]. Likewise, models trained for brain MRI (Magnetic Resonance Imaging) segmentation or diagnostic classification have been shown to capture brain aging markers and vascular comorbidity features not specified in training labels [28,29]. Taken together, these examples demonstrate that deep learning models can identify clinically relevant imaging biomarkers that go beyond human-annotated features, highlighting their potential as hypothesis-generating tools in precision medicine. At the same time, these findings emphasize the need for careful interpretability and validation to confirm that such latent features represent true pathophysiological signals rather than artifacts of the training data.

### 3.2. When Convolutional Neural Networks May Outperform the Human Eye

**Figure 1:**
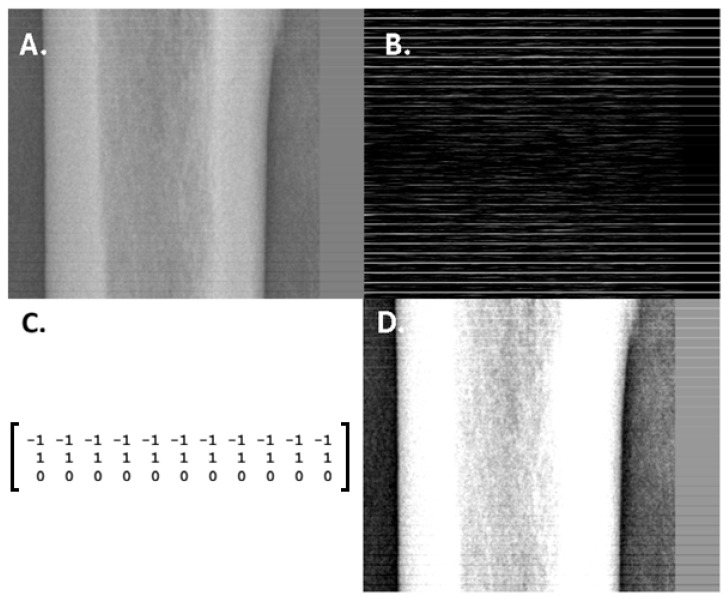
Illustration of the limits of human visual perception and the advantage of convolutional processing in detecting subtle linear structures. (**A**) Original grayscale bone image (8-bit, intensity range 0–255) with simulated horizontal linear fracture patterns. The fractures were generated by superimposing stripe-like artifacts every 10 pixels along the vertical axis, with alternating intensity offsets of +15 and −15 grayscale units relative to the original pixel values. While the stripe pattern remains visible in the peripheral bright and dark regions of the bone, it becomes barely perceptible in the central region due to local contrast masking and limitations of human visual sensitivity. (**B**) Output image after applying a convolutional filter designed to emphasize horizontal intensity discontinuities. The filtering operation enhances the stripe-like structures across the entire image, including regions where the simulated fractures are visually indistinguishable to the human observer in panel (**A**). This demonstrates how convolutional operations can reveal structured patterns embedded in low-contrast or noisy backgrounds. (**C**) Mathematical representation of the convolution kernel used for feature extraction. The kernel consists of three horizontal rows with weights. (**D**) Windowed visualization of the filtered image, illustrating that manual contrast optimization alone is insufficient to fully recover the subtle fracture-like patterns visible in panel (**B**). Despite windowing adjustments, the human eye remains less sensitive to these faint linear features compared with algorithmic convolutional processing.

Numerous studies have reported that convolutional neural networks can detect pathologies in medical images more effectively than human radiologists. This apparent superiority may arise from several factors, one of which is the limitation of human visual perception. In the illustration presented in Figure 1, horizontal linear bone fractures were simulated by adding stripe-like artifacts every 10 pixels, with alternating intensity variations ranging from +15 to –15 relative to the original grayscale values (0–255, 8-bit) (Figure 1A). The stripe pattern is visible in the upper (bright) and lower (dark) regions of the bone but becomes barely perceptible in the central area. Where the human eye no longer distinguishes these subtle changes, convolutional filtering (Figure 1B), mathematically described as a convolution operation (Figure 1C), can still detect the underlying structure. CNNs are built upon such convolutional principles, widely applied in image processing. Consequently, these networks can identify subtle image features that may remain imperceptible to radiologists, even when optimized through windowing (Figure 1D). Available evidence suggests that within-domain reproducibility of AI models is typically strong when training data are large and diverse, whereas cross-institutional and cross-device generalization remains variable. As an example, models predicting demographic or physiological attributes from chest radiographs may preserve signals across datasets but frequently experience performance degradation when transferred to images obtained using different acquisition settings or patient populations [30].

In summary, convolutional neural networks can outperform human visual perception in detecting subtle image features under controlled conditions. Nevertheless, their sensitivity to domain shifts and acquisition variability limits their reliability outside well-defined settings, necessitating continued human oversight.

$$K=\left[\begin{array}{ccccc} -1 & -1 & -1 & \cdots & -1\\ \;\;\;1 & \;\;\;1 & \;\;\;1 & \cdots & \;\;\;1\\ \;\;\;0 & \;\;\;0 & \;\;\;0 & \cdots & \;\;\;0 \end{array}\right],$$

which approximates a directional derivative emphasizing horizontal transitions. The convolution operation is formally defined as

$$(I*K)(x,y)=\sum_{i=-m}^{m}\sum_{j=-n}^{n}I(x+i,y+j)\;K(i,j),$$

where I(x,y) denotes the input image intensity at pixel $\left(x,y\right)$, K(i,j) represents the kernel coefficients, and m,n define the kernel size. This operation effectively computes local intensity differences between adjacent rows, making subtle horizontal structures detectable even when absolute intensity changes are small.

### 3.3. Technical Performance and Real-World Validation

Evidence from recent multicenter analyses supports the conclusion that current AI systems in medical imaging demonstrate high technical performance but limited generalizability across diverse real-world settings. In our previous review (Cancers, 2024) [31], convolutional and transformer-based architectures. for radiological and oncologic imaging achieved mean AUC and F1-scores exceeding 0.9 for lesion detection, segmentation, and tissue classification tasks, confirming their strong discriminative power and internal calibration under controlled conditions. In detection tasks, sensitivity of various AI driven applications ranged from approximately 0.65 to even 1.0. This study [31] highlighted the robust reproducibility of AI-based reconstructions and diagnostic inferences across modalities such as MRI, CT, and digital histopathology, while also noted that performance variability increased markedly when models were applied to external datasets from institutions with differing imaging protocols, equipment, or patient demographics. These findings demonstrate that while AI systems have achieved human-comparable accuracy and calibration, their robustness and generalizability remain contingent on population diversity, data standardization, and retraining procedures. Consequently, effective clinical adoption requires human oversight, continuous validation, and rigorous post-deployment monitoring to ensure safety and reliability in real-world medical practice.

Recent advances in self-supervised pretraining—particularly Transformer and BERT-like architectures—have substantially enhanced downstream model performance in medical imaging [32]. By leveraging large unlabeled datasets, these models learn domain-general feature representations that enable efficient fine-tuning with limited annotated samples, thereby improving sample efficiency and robustness in data-scarce clinical settings. Empirical studies across chest radiography, MRI, and histopathology demonstrate that self-supervised models achieve higher transferability across scanners, institutions, and related diagnostic tasks compared with fully supervised baselines [33].

However, pretraining on large heterogeneous datasets introduces the risk of bias propagation, as demographic or acquisition disparities embedded in the source data may be inherited by the fine-tuned model [34]. To mitigate this, current frameworks increasingly integrate bias-aware sampling, domain adaptation, and balanced pretraining corpora. Overall, self-supervised pretraining represents a pivotal shift toward more generalizable and data-efficient clinical AI models but underscores the continuing need for rigorous bias evaluation before clinical deployment [35].

Interpretability methods in medical imaging AI can be broadly divided into saliency-based, representation-based, and concept-based categories. Attention and gradient-based visualization techniques (e.g., Grad-CAM and integrated gradients) help localize influential image regions and support clinical plausibility assessment, though their outputs can vary with model architecture and input noise [36,37]. Activation atlases and feature clustering approaches probe internal representations, showing that networks often encode anatomically coherent or disease-relevant patterns, yet these analyses remain qualitative and non-standardized [38]. Concept activation methods such as TCAV (Testing with Content Activation Vectors) quantitatively test whether latent features correspond to human-defined clinical concepts (e.g., opacity, mass, and hemorrhage), offering a promising bridge between data-driven and human reasoning [39,40]. However, such techniques depend on curated concept examples and may inherit dataset biases. Overall, explainability tools enhance transparency and scientific understanding but must be interpreted cautiously, as they indicate association rather than causality.

Taken together, these observations demonstrate that contemporary AI systems can achieve high performance in narrowly defined analytical tasks and even extract latent information beyond human perception. At the same time, the task-specific and statistically driven nature of these capabilities limits their applicability to controlled settings, reinforcing the distinction between task automation and full physician replacement. Available results indicate that high technical performance in medical imaging does not guarantee robust real-world generalization. This gap between development and deployment underscores the necessity of external validation, continuous monitoring, and human-in-the-loop integration.

## 4. Barriers to the Full Replacement of Humans by AI Systems in Medical Practice

### 4.1. Regulatory and Legal Issues as Barriers for AI in Medical Practice

One of the most significant issues encountered in the advanced deployment of artificial intelligence in medicine is legal and regulatory governance. A first question is whether completely delegating the care of human health—understood as an individual’s most important asset—to a machine is morally acceptable [41]. Where an AI system acts on a patient, an ongoing regulatory debate concerns who should be held responsible for the outcomes of such actions. It should be noted that a machine—even a highly autonomous one—remains a human artifact and is managed by humans; however, it is still unclear who, and to what extent, should bear the consequences of machine–patient interactions [42].

Despite varying degrees of autonomy assigned to systems, liability for the consequences of the actions of machines and AI systems is not directly “scaled” in EU law according to the autonomy level declared in the CE marking. The European Parliament, in its resolution of 16 February 2017, nevertheless advocated that, when allocating liability, the degree of system autonomy and the scope of instruction and training should be considered—such that the greater the autonomy/learning capacity, the greater the responsibility of the entity exercising oversight over the “training”—with responsibility ultimately remaining with a human [43,44]. Under the forthcoming Artificial Intelligence Act (Regulation (EU) 2024/1689), the degree of autonomy declared in an AI system’s CE marking directly determines its regulatory obligations and liability profile. Assistive systems must operate under continuous human supervision, with clinicians retaining responsibility for patient outcomes, whereas systems claiming greater autonomy are subject to markedly stricter regulatory and liability requirements.

This distinction defines the current regulatory ceiling for medical AI: CE-marked systems are legally designed to support clinicians, not to act as independent decision-makers. As a result, full physician replacement remains incompatible with existing regulatory and ethical frameworks.

Importantly, CE marking reflects the declared intended use and risk classification of a system rather than granting independent legal agency or responsibility to the algorithm itself.

A human, faced with uncertainty regarding how to save another person’s life, can make a provisional (revised) decision or decide to seek consultation. What matters here is the ability to couple reflexive responses to stimuli with analytic, logical reasoning that takes place within human higher-order cognitive reasoning [45]. A machine operates by means of an algorithm which, although it may be modified within the layers of deep networks, follows directions of operation and modifiability—so called “bias”—that are always influenced by the information provided by humans. Machine operation is always a human artifact (both as to its physical substrate—hardware—and as to the computer program—software), so there is ultimately a diffuse responsibility of developers that is difficult to delineate and, consequently, to enforce; to date, this remains an unresolved legal issue [46].

### 4.2. Human–Machine Interaction Gaps

A central barrier to full physician replacement lies in the fundamental gap between human and machine interaction with patients and the physical world. This gap reflects well-established limits of artificial intelligence related to embodiment, sensorimotor grounding, and real-world interaction [47]. It encompasses the absence of physical examination, limited multisensory perception, lack of embodied cognition, and restricted manual and procedural skills in current AI systems. While these limitations are often discussed independently, they collectively represent a shared structural constraint: contemporary AI lacks direct, sensorimotor engagement with patients and therefore cannot reproduce the interactive and embodied nature of clinical practice [48].

Despite their ability to analyze imaging data and other digitized inputs with high precision, AI systems remain unable to perform or meaningfully substitute for physical examination. Diagnostic information derived from palpation, tissue stiffness, olfaction, posture, gait, and fine motor interaction plays a critical role in clinical reasoning but is inaccessible to purely algorithmic systems [49,50]. Although emerging technologies such as wearable sensors, haptic interfaces, and robotic platforms attempt to approximate selected sensory modalities, systematic reviews indicate that these systems currently lack the integration, standardization, and clinical validation required for routine bedside use [51,52]. Consequently, such technologies remain complementary rather than substitutive to human clinical assessment.

The human–machine interaction gap becomes even more pronounced in procedural and interventional medicine. Clinical practice extends beyond information processing to include a wide range of manual activities, from endodontic procedures and endoscopy to complex surgical actions. Human clinicians continuously integrate visual, tactile, and proprioceptive feedback, as well as an implicit understanding of physical constraints such as gravity and resistance, to adapt their actions in real time [53]. In contrast, current AI systems and robotic platforms lack this level of embodied intelligence and contextual awareness, a limitation classically described by Moravec’s paradox, whereby sensorimotor tasks that are intuitive for humans remain exceptionally difficult for machines [54,55].

Collectively, these interaction- and embodiment-related constraints demonstrate that the limits of physician replacement are not merely technological but structural. Until AI systems can engage patients through rich, bidirectional physical interaction and assume responsibility for embodied clinical action, their role will necessarily remain assistive and augmentative, operating under continuous human supervision [56].

### 4.3. Statistical and Out-of-Distribution Generalization Barriers in Medical AI

In radiology, which stands at the forefront of AI-assisted medical disciplines, there is also an emerging problem of lesions for which visual representation is unknown or poorly known to the machine. Highly precise recognition and measurement of lesions is strictly limited to known patterns. If a cluster of points which represents a region with affinities (shape and intensity) is unknown to the machine, it might be not able to recognize it. If the program’s assumptions compel the issuance of an opinion, it may be erroneous [57]. In ambiguous situations, under typical program assumptions, a finding in an imaging study is either omitted or flagged but left without a definitive response, placing the patient in a potentially unsafe situation. This limitation is not theoretical but has been repeatedly observed in clinical translation. For example, an AI model trained exclusively on imaging data from a single high-volume tertiary center—such as Memorial Sloan Kettering Cancer Center (MSKCC)—may fail when applied in a community hospital setting. Differences in scanner type, acquisition parameters, contrast agent protocols, patient demographics, and local disease prevalence can all cause the algorithm to encounter patterns that fall outside its training distribution [58]. Such shifts often result in substantial drops in diagnostic accuracy or spurious classifications, even when the same pathology is present. This phenomenon exemplifies the out-of-distribution (OOD) problem: models that perform exceptionally well in development settings may degrade or behave unpredictably in real-world, heterogeneous clinical environments. Such failures are commonly driven by differences in scanner vendors, acquisition protocols, reconstruction parameters, patient demographics, and disease prevalence between institutions. Standard mitigation strategies include multi-center training, external validation, continuous performance monitoring, and mechanisms allowing the system to defer to human experts when uncertainty is high. Consequently, external validation across diverse populations and institutions remains an essential prerequisite for any AI system intended for clinical deployment.

Challenges in the introduction of “mature purely analytical systems” in the real world of contemporary healthcare are reflected in domain-specific analyses such as the recent review “Progress and challenges of artificial intelligence in lung cancer clinical translation” [59]. Although AI algorithms in lung cancer screening have demonstrated exceptionally high diagnostic performance (AUC > 0.90 in several studies), the review highlights persistent obstacles to clinical translation—most notably data and model bias, lack of cross-institutional standardization, limited reproducibility, and failures of generalizability when applied to heterogeneous patient populations or imaging systems. These issues underscore that even in high-stakes fields with mature datasets, AI remains constrained by regulatory, technical, and ethical boundaries that preclude autonomous operation. Consequently, the foreseeable future of medical AI continues to center on augmentation, with clinicians retaining decisive responsibility for interpretation, context integration, and patient safety [60].

### 4.4. Embodied Cognition as a Structural Boundary to Physician Replacement

There are many situations in which machine knowledge is insufficient because it lacks the full spectrum of information that physicians obtain through direct patient contact. Although emerging technologies such as wearable sensors, haptic interfaces, and robotic systems attempt to approximate selected sensory inputs, they currently lack the standardization, integration, and clinical validation required to replace bedside examination. Consequently, these technologies remain complementary rather than substitutive to human clinical assessment. At the current stage of technological development, machines are unable to physically examine patients. While AI systems can process large volumes of “theoretical” data derived from laboratory tests, imaging studies, medical history, and demographics, they lack access to the rich dataset generated through direct human interaction with the patient [61,62].

A critical example of this limitation is palpation, understood as the assessment of tissue stiffness. Despite ongoing research efforts, there are currently no clinically adaptable systems capable of physically touching patients in a standardized manner to evaluate tactile features that are essential for diagnostic reasoning [63]. Similarly, computer-based diagnostic systems lack olfactory information and standardized assessment of patient gait and movement. Odor cues and patterns of movement can provide experienced clinicians with clinically relevant information that cannot be readily inferred from numerical or imaging data alone [64]. As a result, a substantial portion of diagnostic information long established in medical practice—particularly in internal medicine—cannot be incorporated into algorithmic decision-making and may be lost during AI-based patient evaluation [65].

When considering whether machines could relieve or replace physicians, it is essential to account for persistent physical and procedural barriers. Medicine is not limited to conversation and prescription generation; it encompasses a broad range of manual activities, from dental procedures to complex surgical interventions. Any discussion of physician replacement must therefore consider this full scope of practice. At present, machine competence is strongest in language-based and pattern-recognition tasks [66]. Although language is often regarded as a hallmark of human intelligence, it represents a comparatively tractable domain for machine learning because it relies on a finite set of symbolic tokens. With sufficient computational resources, large language models can perform complex operations over this symbolic space. However, such systems do not provide genuine understanding of the physical world, as visual and tactile stimuli are processed primarily in descriptive or abstracted form rather than through direct interaction [67].

Human capabilities, shaped by millions of years of evolution, integrate perception across vision, touch, balance, and an implicit understanding of physical forces such as gravity, enabling the effective execution of complex manual tasks. Human common sense and contextual understanding arise from embodied cognition, which combines multisensory inputs to construct an internal causal model of the physical world—capacities that current AI systems do not possess [68,69]. This asymmetry, which has important practical implications in medicine, is encapsulated by Moravec’s paradox: perceptual and motor tasks that are trivial for humans remain profoundly difficult for machines [70,71].

Addressing these limitations will be essential for the development of next-generation embodied AI systems capable of integrating perception, reasoning, and motor control in a biologically inspired, non-language-driven manner. To date, despite extensive experimental efforts, AI systems continue to operate primarily on symbolic or descriptive representations of the physical world. Although attempts have been made to encode real-world stimuli through language-based models [72,73,74], such approaches have not yielded a level of understanding sufficient to support reliable physical action in medical settings [75,76]. Consequently, the replacement of physicians—particularly in primary care and surgical specialties—would require fully autonomous robotic systems endowed with generalizable embodied intelligence, technologies that remain far beyond current feasibility and the temporal horizon of this review.

Similarly, large language models (LLMs) exhibit important limitations. LLMs are inherently stochastic and probabilistic systems that generate outputs by selecting the most likely continuation from a set of candidate responses, operating under a paradigm of forced response generation [77]. When exposed to underrepresented, ambiguous, or unfamiliar contexts, these models may produce confident but incorrect outputs—a phenomenon commonly described as hallucination or confabulation. Such errors arise not from reasoning failure in a human sense but from statistical pattern completion in the absence of sufficient contextual grounding. In clinical contexts, this limitation necessitates the use of guardrails such as retrieval-augmented generation, explicit citation of source material, refusal mechanisms for uncertain queries, and mandatory human verification. Without such safeguards, confident but incorrect outputs may pose significant patient safety risks. Humans solve such problems using contextual experience—the “I’ve heard/seen this somewhere” heuristic—by reexamining sources or seeking consultation to reach consensus, thereby enabling provision of the most probable answer. In professional practice, risk-taking behaviors that consist of providing an answer at all costs are less likely [78].

The scope of potential AI substitution versus augmentation can be defined according to the nature of the clinical task and its dependence on embodied skills or contextual reasoning. Substitution is most plausible in domains characterized by standardized, high-volume data and well-defined diagnostic endpoints—such as radiology, histopathology, and selected branches of cardiology and dermatology—where convolutional neural networks and transformer-based architectures have achieved near-human performance in image detection, segmentation, or pattern classification (AUC/F1-scores typically exceeding 0.9) [79]. These applications involve discrete, reproducible tasks that can be validated statistically and are amenable to automation under physician supervision.

Conversely, augmentation rather than replacement is expected to dominate in fields requiring multimodal integration, physical examination, or continuous interpersonal interaction—such as internal medicine, primary care, surgery, psychiatry, and emergency medicine. In these specialties, AI can support decision-making, documentation, triage, and image interpretation, but the decisive clinical judgment, manual dexterity, and empathic communication required for patient safety remain human-dependent. Accordingly, the limits of substitution are defined less by algorithmic capability than by the current absence of embodied intelligence and the ethical, legal, and contextual dimensions of clinical care.

Collectively, these legal, ethical, and epistemic constraints illustrate that the barriers to physician replacement are structural rather than merely technological. Current regulatory frameworks and responsibility models explicitly presuppose human oversight, thereby delimiting the permissible scope of medical AI autonomy.

## 5. Machine Learning in Radiology—Limitations Implied by Statistics

These limitations are especially evident in radiology, one of the most AI-intensive fields, which serves as a use case here. Statistics as such, and, consequently, statistical inference, have their own specific characteristics. First, they concern mass phenomena [79]. This means, among other things, that the sampling procedures are used to collect information that serves as the basis for statistical inference [80]. These methods are widely used to support decision-making in both clinical and organizational settings. However, statistical inference is inherently imperfect, and machine learning systems are fundamentally based on probabilistic reasoning. Even an accuracy of 90%—a level that is rarely achieved in real-world medical AI applications—implies incorrect decisions in approximately 100 out of every 1000 cases.

While such performance may appear satisfactory from a statistical standpoint, its implications in clinical practice are far more serious. Each percentage point of error corresponds to real patients who may experience harm or loss of life. Because human dignity and subjectivity form the ethical foundation of healthcare, each patient must be treated as an individual rather than as a statistical outcome [81]. An algorithm trained using statistical methods cannot provide such an approach; individual treatment of a person can only be provided by a person. Nevertheless, AI can become a powerful tool in supporting medical personnel. Radiology is a useful example here because it combines high-volume standardized data with direct clinical consequences of error.

Radiology is one of the earliest and most intensive fields for the clinical deployment of AI. Deep learning systems, particularly convolutional neural networks, can achieve remarkable accuracy in detecting lesions and recognizing patterns in medical images. At the same time, radiology highlights some of the most fundamental limitations of AI in medicine—limitations that are directly relevant to broader adoption across various specialties. A central challenge lies in the statistical nature of machine learning. AI systems perform well on typical cases that are abundantly represented in training datasets but often fail when confronted with rare, atypical, or out-of-distribution presentations. As discussed in Section 2.3, this general challenge becomes particularly visible in radiology. During validation, models have been shown to misclassify even slight deviations from the training distribution, including images with small pixel-level perturbations or subtle variations in acquisition parameters and image quality [82,83]. In practice, such deviations may arise from differences among scanner vendors, protocols, reconstruction settings, positioning, or motion artifacts. These errors highlight the difficulty of generalizing beyond narrowly defined statistical norms. Furthermore, a statistical approach to training AI systems overlooks the detection of information hierarchy, which is crucial in certain instances of medical data [84]. For instance, the same subtle finding may have different significance depending on anatomical location, clinical question, or comorbidities.

Human radiologists integrate spatial information (e.g., symmetry and anatomical location), patient history, age, the hierarchical character of anatomical structures, and comorbidities when interpreting subtle differences in images. AI, by contrast, reduces images to pixel–label associations and is blind to context unless explicitly encoded or provided. This limitation is particularly acute for unusual conditions—such as rare tumors or vascular anomalies that are poorly represented in training data and thus not reliably recognized [85]. Recent work has explored multimodal architectures that combine imaging and clinical data. These approaches aim to provide a more holistic representation of the patient, but studies consistently report that generalization to rare or atypical cases remains limited [85,86]. Similarly, XAI methods—such as biomarker activation maps in MRI—help visualize why an algorithm reached a particular decision and reveal when it focuses on irrelevant features [87]. Yet, these approaches, while increasing transparency, do not fully resolve the problem of statistical blindness—understood here as strong performance on in-distribution data combined with fragility to rare or atypical cases.

From a practical perspective, these limitations suggest several directions for enhancing the robustness and safety of AI in radiology. First, models trained predominantly on typical cases require more diverse, multi-center datasets in which rare diseases and atypical presentations are deliberately enriched, supported by careful data curation and appropriate augmentation strategies. In addition, methods for uncertainty estimation and explicit detection of out-of-distribution inputs can enable AI systems to “abstain” and defer to the radiologist when confidence is low, rather than producing overconfident but unreliable predictions. Domain adaptation techniques and continuous revalidation across different scanners, protocols, and patient populations are likewise essential for mitigating performance drops when imaging conditions change [87,88]. Overall, this reinforces the conclusion that, even in radiology, safe deployment relies on human-in-the-loop oversight rather than autonomous decision-making, a point further developed in Section 5.

Additionally, the integration of AI tools into clinical workflows is crucial (see Section 1). To minimize cognitive load and reduce automation bias, AI outputs should be embedded within existing PACS/RIS interfaces and presented in a way that allows radiologists to retain primary responsibility for interpretation. In this context, AI is most appropriately used as an augmentative tool rather than a substitute for clinical judgment. XAI techniques, including heat maps and other visual overlays, can serve as audit tools that help radiologists understand when to trust or disregard algorithmic suggestions [89,90].

Radiology, therefore, exemplifies the phenomenon often termed “statistical blindness”: strong performance on well-known data, coupled with fragility when faced with variation [91,92,93,94]. While explainable AI enhances system transparency and clinician trust, it does not resolve the underlying problem of limited generalization. Importantly, radiology also illustrates the enduring gap between algorithmic pattern recognition and human clinical reasoning, which relies on intuition, contextual judgment, and flexible decision-making. It demonstrates how technical issues—such as overfitting to statistical patterns and failure to integrate clinical context—intersect with the broader barriers to adoption discussed in the following section. This understanding is crucial for the future of radiology and the wider medical field, ensuring that AI enhances the work of medical professionals rather than replaces it.

In summary, radiology exemplifies both the strengths and limitations of statistical learning in medicine: while AI systems excel in high-volume, standardized tasks, their statistical nature makes them fragile in rare, atypical, or context-dependent cases. This reinforces the necessity of physician judgment in clinically consequential decisions.

## 6. Reliability and Trust in AI-Based Diagnosis and the Role of Physicians

Full automation of the diagnostic process may raise concerns among both physicians and patients. This is primarily linked to the lack of explainability of models created by neural networks, as it may turn out that a diagnosis is based on an incidental input value or on some other feature that merely happens to correlate with the disease process—something that cannot be verified.

The acquisition context of the evaluated studies may also prove crucial: it is unclear how a trained network will behave if it is provided data from, for example, a new device that may have its own idiosyncratic characteristics. There is no certainty as to what is advisable in such cases—one must perform a series of tests and, possibly, retrain the network with the new data. These issues require the entire development team to be involved in assessing the quality of such diagnoses.

### 6.1. Hallucinations and the Necessity of Physician Oversight

One common, frequently encountered problem is hallucinations—or, more precisely, confabulations [95]. They arise as modifications of outputs based on the network’s statistical belief about what the model should look like (i.e., what “fits”). This is particularly harmful in diagnostic image analysis. Such situations occur very often during MRI reconstructions. Networks then attempt to impose their own solution, which is either based on the most prevalent state (i.e., no pathology), as shown in [96], where reconstructions effectively concealed meniscal tears or subchondral osteophytes. They may insert other structures, such as false vessels [97]; they may even suggest pathology where none exists by generating structures that resemble real brain lesions—e.g., a cleft of cerebrospinal fluid on MRI [98]; or they sometimes remove structures, such as the total loss of some papillary muscles in [99] or loss of small peripheral hepatic vessels [100]. Physicians therefore argue that they must remain the final arbiters when assessing whether AI-detected fractures are genuine [100]. In medical AI, hallucinations manifest differently depending on the underlying architecture.

In image reconstruction using convolutional neural networks, hallucinations (can be referred as visual confabulations) often take the form of false or missing anatomical features, such as non-existent blood vessels, concealed meniscal tears, or the removal of genuine structures like papillary muscles. These artifacts typically result from statistical bias or over-regularization.

In contrast, large language models used for automated reporting may generate textual hallucinations, producing seemingly plausible but unsupported diagnostic statements. Both forms of textual hallucinations and visual confabulations threaten clinical reliability and reinforce the necessity of sustained physician oversight.

Based on the work of Mirzadeh et al. [101], hallucinations and visual confabulations appear to be an inherent characteristic of large language models because their predictions rely on statistical pattern matching rather than genuine symbolic or logical reasoning. When confronted with gaps or ambiguities, LLMs tend to fill in missing steps with plausible-sounding approximations instead of verifiable deductive processes, which naturally leads to confident but incorrect outputs.

Overall, issues such as hallucinations, adversarial vulnerability, and automation bias demonstrate that trust in AI-based diagnosis cannot be grounded in performance metrics alone. Sustained physician involvement remains essential to safeguard clinical reliability and patient safety.

**Figure 2:**
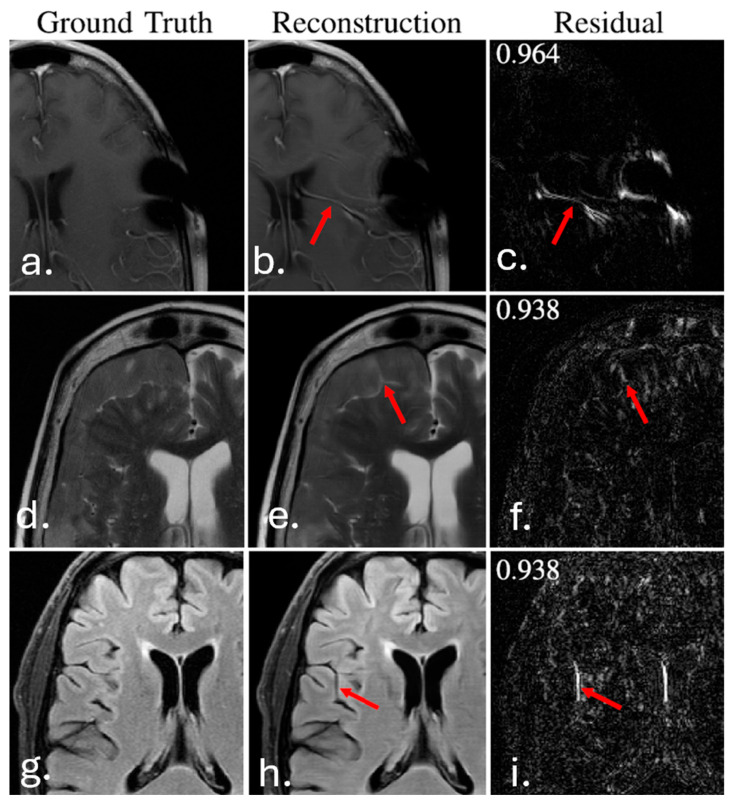
Qualitative comparison of ground-truth images, reconstructed outputs, and residual error maps across acceleration and transfer-learning conditions. Rows represent (**a**–**c**) 4× acceleration, (**d**–**f**) 8× acceleration, and (**g**–**i**) transfer-learning reconstruction. Columns show the ground truth, model reconstruction, and the corresponding residual map (reconstruction—ground truth), with the structural similarity index (SSIM) displayed in the upper-right corner of residual images. Red arrows highlight regions where subtle structural deviations, smoothing, or incomplete recovery of fine pathological details are most prominent. Residual images demonstrate increasing error patterns with higher acceleration factors, while transfer-learning reconstruction maintains stable performance with localized discrepancies in the vascular structures representation. The figure is from [97], Figure 6, licensed under CC BY-ND 4.0.

To illustrate the clinical significance of this problem, representative examples of serious confabulated features were presented: Figure 2 [97].

### 6.2. Heightened Sensitivity to Perturbations in AI-Generated Diagnosis

**Figure 3:**
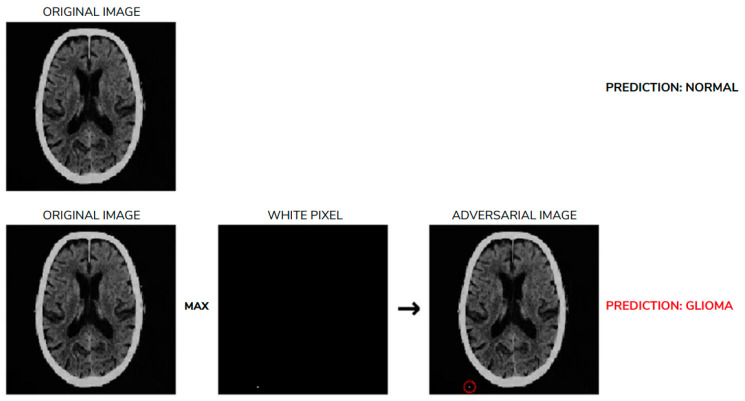
Illustration of a one-pixel adversarial attack in medical image classification. The upper panel shows the original axial brain image correctly classified by the neural network as normal. In the lower panel, a single-pixel perturbation (white pixel with maximum intensity, indicated schematically in the center and highlighted by a red circle in the image) is introduced at a location outside the anatomically relevant brain structures. Despite the minimal and visually imperceptible modification, the resulting adversarial image leads the model to misclassify the case as glioma. This example demonstrates the high sensitivity of deep neural networks to localized pixel-level perturbations and highlights the potential vulnerability of AI-based diagnostic systems to adversarial noise, even when changes occur outside the region of clinical interest. The figure is based on the study by Tajak et al. (2025) [103], illustrating the implications of adversarial robustness for patient safety in medical imaging. Licensed under CC BY 4.0.

A separate issue is adversarial attacks on data. In theory, this involves forcing an alternative diagnosis by overlaying, for example, patches on the image or altering its content. Even more alarming are one pixel attacks [102], in which changing a single pixel can alter the network’s prediction. This is not farfetched, because such a pixel change may arise as interference (noise), as in MR imaging when acquisition time is reduced. In 2025, it was shown [93] that the number of such uncertain pixels may reach, depending on the network architecture used, even about 100 per image; what is particularly troubling is that these may be pixels located outside the human body (Figure 3). In such cases, the participation of a radiologist as an arbiter is necessary.

### 6.3. Disappointed Hopes

We should also discuss the disappointed hopes in medicine—an issue rarely addressed today in the expectation that the steady development of AI tools will overcome temporary shortcomings. For example, it turned out that the accuracy of machine learning algorithms for predicting suicidal behavior is too low to be useful for screening or for prioritizing high-risk individuals for interventions [104]. An even more worrisome aspect of AI is the gradual deskilling of physicians, as shown in [105], where the adenoma detection rate of standard non-AI-assisted colonoscopy decreased significantly from 28% before to 22% after discontinuing the use of AI assistance tools for polyp detection. The phenomenon of gradual deskilling represents a tangible risk of sustained AI integration in clinical workflows. Prolonged reliance on algorithmic recommendations may attenuate physicians’ ability to independently interpret findings, perform complex procedures, or critically evaluate automated outputs. Such erosion of experiential skills could compromise diagnostic vigilance and reduce adaptability in atypical or high-risk cases. Addressing this risk requires deliberate strategies in training and continuous professional development to ensure that physicians remain active, reflective decision-makers rather than passive overseers of AI outputs.

In the context of unmet expectations, it is important to highlight experiments in which the anticipated advantages of neural networks were not fully realized. For example, in our previous study [106], we demonstrated that while age estimation achieved comparable accuracy using both AI-based and classical approaches, sex differentiation yielded notably poorer results for deep learning models—69% accuracy with texture analysis compared to only 59% for convolutional networks. Similarly, in another study [107], neural networks performed well in identifying extreme joint conditions on the Kellgren–Lawrence scale (grade 0—normal; grade 4—advanced, complete osteoarthritis) yet failed to reliably detect subtle changes in intermediate stages of disease progression. These findings emphasize that, despite significant progress, deep learning systems may still struggle with nuanced or borderline cases where visual cues are subtle and contextual reasoning remains essential [108].

**Table 2:** Representative performance metrics of selected artificial intelligence applications across clinical domains. Reported values summarize typical performance ranges described in the literature and are not intended as direct algorithmic comparisons.

Clinical Domain	AI Task	Representative System/Study (As cited)	Dataset/Setting	Key Performance Metrics	Interpretation in Context of Physician Replacement
Radiology (CT)	Intracranial hemorrhage detection	Titano et al., 2018 [2]	Multicenter CT scans	Sensitivity ≈ 0.90–1.00; AUC > 0.90	High sensitivity supports triage and prioritization; false positives and out-of-distribution risk necessitate radiologist oversight
Radiology (CT)	Pulmonary embolism detection	Ardila et al., 2019; FDA-cleared triage tools [8]	CT pulmonary angiography	Sensitivity up to ~0.95; AUC ≈ 0.90–0.94	Effective for workflow acceleration; not validated for autonomous diagnosis
Mammography	Breast cancer detection	Rodríguez-Ruiz et al., 2019 [3]; Salim et al., 2020 [4]	Screening mammography	AUC ≈ 0.94–0.97; sensitivity comparable to expert radiologists	Demonstrates near-expert performance in a narrowly defined task, supporting second-reader or triage use
Radiology (X-ray)	Pneumonia and lung pathology detection	Zech et al., 2018 [30]	Single- vs. multi-institution datasets	AUC > 0.90 (internal validation); marked performance drop on external data	Illustrates limited generalization and dependence on training distribution
MRI reconstruction	Accelerated image reconstruction	fastMRI challenge (Knoll et al., 2020, Muckley et al. 2020) [96,97]	Multivendor MRI datasets	SSIM often > 0.95; localized hallucinations reported	High quantitative image quality; hallucinated or missing structures preclude unsupervised clinical use
Large language models	Medical examinations and diagnostic reasoning	Kung et al., 2023 [23]	USMLE-style examinations	Accuracy > 0.85 on MCQs; misdiagnosis rate ~10–15% in case analyses	Strong associative reasoning; error rate incompatible with autonomous clinical responsibility
Colonoscopy	Polyp and adenoma detection	Budzyń et al., 2025 [105]	Multicenter real-world endoscopy	Improved detection during AI assistance; reduced vigilance after withdrawal (20%)	benefit with documented risk of physician deskilling
Sepsis prediction	clinical deterioration prediction	Spittal et al., 2025 [104]	EHR-based predictive models	AUROC moderate; limited clinical benefit	Illustrates gap between predictive performance and outcome improvement

Reported metrics originate from heterogeneous datasets, study designs, and clinical tasks and are therefore not directly comparable. The table is intended as a concise overview illustrating that high quantitative performance in narrowly defined tasks does not equate to safe or autonomous physician replacement.

**Table 3:** Representative performance metrics reported for artificial intelligence applications across major radiological modalities, based on studies discussed in the editorial topic AI in Medical Imaging and Image Processing [108]. The table summarizes typical ranges of diagnostic performance (e.g., AUC, F1-score, and sensitivity) rather than providing a head-to-head comparison of algorithms.

Imaging Modality	Clinical Task	AI Approach (as Reported)	Representative Performance Metrics *	Clinical Interpretation
MRI	Brain tumor detection and classification	Convolutional neural networks (CNNs)	AUC ≈ 0.88–0.97; F1-score ≈ 0.88–0.97	Near-expert performance in well-defined neuro-oncologic tasks under controlled conditions
MRI	Accelerated image reconstruction	Deep learning reconstruction networks	SSIM > 0.95; PSNR improvement reported	High quantitative image quality, with a documented risk of hallucinated or missing structures
CT	Lung cancer screening and nodule detection	3D CNN-based detection models	AUC ≈ 0.90–0.94	Effective for detection and triage; performance sensitive to dataset composition and domain shift
CT	Intracranial hemorrhage detection	CNN-based classification and triage systems	Sensitivity ≈ 0.90–1.00; AUC > 0.90	High sensitivity supports emergency triage but does not eliminate false positives
X-ray (Chest)	Pneumonia, COVID-19, and tuberculosis detection	CNN architectures (e.g., ResNet, VGG)	F1-score ≈ 0.95–0.99 (internal validation)	Excellent in-distribution performance with reduced robustness across institutions
Mammography	Breast cancer detection	Deep learning CAD systems	AUC ≈ 0.94–0.97	Comparable to expert radiologists in screening settings; typically used as a second reader
Ultrasound	Musculoskeletal and joint assessment	CNN-based segmentation and classification	F1-score ≈ 0.84–0.91	Promising results, but limited generalization and operator dependence
Histopathology (WSI)	Tumor classification and mutation prediction	CNN and weakly supervised deep learning	AUC ≈ 0.90–0.99 (task-dependent)	Strong performance in subtype classification; translation limited by domain shift
Multimodal (Imaging + text)	Diagnostic reasoning and reporting	LLM-assisted systems	Misdiagnosis rate ≈ 0.10–0.15	Useful for documentation and decision support, not autonomous diagnosis

* Reported metrics originate from heterogeneous datasets, study designs, and clinical tasks and are therefore not directly comparable.

## 7. Discussion

The central question addressed in this review—whether artificial intelligence can replace physicians in the near future—cannot be answered by performance metrics alone. To contextualize these arguments, Table 4 summarizes representative studies across clinical domains, highlighting typical performance levels alongside recurring failure modes and structural limitations relevant to physician replacement. While numerous studies report high accuracy of AI systems in narrowly defined tasks, we argue that technical proficiency is neither equivalent to, nor sufficient for, physician replacement. Representative examples of AI performance across clinical domains, together with their key limitations in the context of physician replacement, are summarized in Table 2.

### 7.1. Accuracy Is Not Equivalence: Statistical Performance Versus Clinical Responsibility

A recurring theme in the contemporary AI literature is the emphasis on high diagnostic accuracy, often expressed through aggregate metrics such as AUC, sensitivity, or F1-score [6,109]. From a statistical perspective, these values may appear compelling. From a clinical perspective, they conceal a fundamental asymmetry: medicine is practiced at the level of individual patients, not populations [110]. Even a system with 90% accuracy necessarily implies systematic harm to a nontrivial number of individuals. Physicians, by contrast, are ethically and professionally obligated to treat each diagnostic failure as unacceptable, not statistically tolerable [41].

We therefore argue that the statistical foundations of machine learning introduce an irreducible tension with clinical accountability. AI systems optimize expected performance across datasets, whereas clinicians operate under a normative framework centered on individual responsibility, contextual judgment, and moral agency. This divergence explains why high technical performance has not translated into acceptance of autonomous clinical decision-making and why, in practice, AI remains embedded within human-in-the-loop workflows [109,111].

Thus, statistical accuracy alone is insufficient to justify autonomous clinical decision-making, as clinical responsibility is fundamentally individual rather than probabilistic.

### 7.2. Generalization and the Problem of the Unknown

Our synthesis further highlights that AI systems remain fundamentally constrained by their dependence on training distributions. While convolutional neural networks demonstrate impressive reproducibility within known domains, their vulnerability to out-of-distribution inputs represents a critical safety limitation [30,88]. Rare diseases, atypical presentations, and shifts in acquisition protocols are not edge cases in medicine—they are routine clinical realities [34].

We emphasize that this limitation is not a temporary implementation flaw but a structural property of data-driven models [57]. In contrast to physicians, who can explicitly recognize uncertainty, seek consultation, or suspend judgment, AI systems tend to produce outputs even when underlying assumptions are violated [104]. This asymmetry reinforces the necessity of physician oversight and undermines claims that AI systems can assume autonomous diagnostic responsibility in real-world clinical environments [112].

### 7.3. Embodiment as a Non-Negotiable Dimension of Medical Practice

A key original contribution of this review is the explicit integration of embodied cognition into the debate on physician replacement. Medical diagnosis is not reducible to image interpretation or text analysis. Physical examination—palpation, assessment of movement, posture, tenderness, smell, and manual interaction—constitutes a substantial and irreplaceable component of clinical reasoning [41,71].

Current AI systems operate almost exclusively on symbolic or pixel-based representations and lack direct access to these sensory modalities. Although experimental robotic and mobile systems for patient interaction have been explored, they remain fragmented, non-standardized, and clinically immature [63,65]. We argue that this embodiment gap is not incidental but fundamental: without grounded sensorimotor understanding, AI systems cannot replicate the causal, adaptive reasoning that underpins everyday medical practice [70,113]. This limitation sharply delineates the boundary between task automation and physician substitution. Accordingly, the absence of embodied cognition represents a fundamental barrier to physician replacement that cannot be resolved by advances in pattern recognition alone.

Recent progress in multimodal and foundation model research indicates a potential pathway toward mitigating some of the generalization and data-integration limitations identified in this review. By integrating imaging, clinical, and molecular information within shared representation spaces, these models seek to capture broader clinical context than task-specific algorithms [114]. Emerging evidence suggests that such foundation models may improve transferability and robustness across domains; however, they remain in early stages of clinical validation. Importantly, these advances do not eliminate fundamental challenges related to embodiment, physical examination, and accountability and therefore do not alter the conclusion that current AI systems primarily function as assistive tools rather than autonomous clinical agents.

### 7.4. Legal and Ethical Constraints Are Not Externalities

Discussions of physician replacement often treat regulation and liability as external obstacles that will eventually be resolved. Our analysis suggests the opposite: current legal frameworks actively encode the assumption that AI systems are assistive rather than autonomous [43,44]. Under European and comparable regulatory regimes, responsibility for clinical decisions remains explicitly human, regardless of algorithmic sophistication [1,42].

This is not merely a regulatory lag but a reflection of unresolved ethical questions surrounding accountability, trust, and moral agency [41]. A system that cannot bear responsibility cannot replace a professional whose defining role includes precisely that responsibility. From this perspective, law and ethics do not trail technological progress; they define its permissible scope [115].

### 7.5. Augmentation, Not Replacement, as the Clinically Coherent Trajectory

Taken together, these considerations lead us to a clear conclusion: the most plausible and ethically coherent future for AI in medicine is augmentation rather than replacement [116]. AI systems already demonstrate substantial value in automating well-defined, high-volume tasks—such as image triage, measurement, documentation drafting, and decision support—thereby reducing workload and variability [6,31].

**Figure 4:**
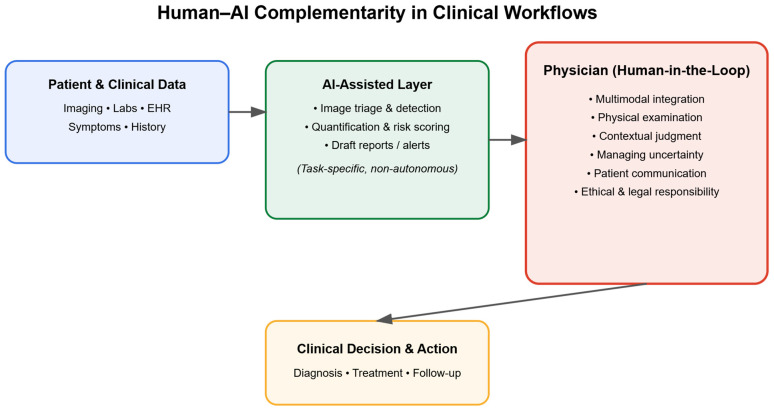
Conceptual model of human–AI complementarity in clinical workflows. Artificial intelligence systems operate as task-specific, assistive tools that preprocess and analyze selected data streams (e.g., imaging, laboratory results, and electronic health records). The physician integrates AI outputs with physical examination, clinical context, ethical judgment, and legal responsibility to reach the final diagnostic or therapeutic decision. The diagram illustrates why current AI systems augment rather than replace physicians in real-world clinical practice.

Integration of AI into clinical care does not diminish the physician’s role; rather, it sharpens it. As automation expands, the physician’s responsibility increasingly shifts toward oversight, integration of heterogeneous data, management of uncertainty, patient communication, and accountability for outcomes [112,113]. These functions are not residual tasks awaiting automation; they define the profession itself (Figure 4).

### 7.6. Relative Clinical Maturity of Imaging AI and Large Language Models

Large language models warrant distinct consideration because their apparent clinical competence derives primarily from linguistic fluency rather than embodied or causal understanding. High performance on medical examinations and written communication tasks reflects probabilistic pattern completion over text, not real-time clinical reasoning under uncertainty. Consequently, while LLMs may approximate physician performance in documentation, education, and asynchronous communication, they remain unsuitable for autonomous diagnostic or therapeutic decision-making. Their role therefore aligns with augmentation rather than substitution, reinforcing the broader conclusions drawn from imaging-based AI systems.

Importantly, these limitations become more pronounced in real clinical workflows, where LLMs are exposed to incomplete, evolving, and heterogeneous information over time. In longitudinal care settings, LLMs may propagate early inaccuracies across subsequent notes or recommendations, reinforcing erroneous assumptions rather than revising them as new evidence emerges. Hallucinations—confident generation of unsupported or incorrect clinical statements—pose particular risks in documentation, handover summaries, and patient-facing communication, where errors may remain undetected and influence downstream clinical decisions. In addition, because LLMs inherit statistical regularities from their training data, they may amplify existing biases embedded in clinical language, guidelines, or population representation, leading to systematic disparities when deployed at scale.

These limitations are consistent with broader critiques of foundation models in medicine, which emphasize that impressive general-purpose performance does not translate directly into clinical reliability, safety, or accountability. As discussed by Muneer et al. [117], foundation models often blur task boundaries, exhibit limited uncertainty awareness, and shift failure modes from overt errors to subtle, workflow-level risks that are difficult to detect, audit, or regulate. In the case of LLMs, this manifests as plausible but incorrect narrative outputs that may appear clinically coherent while lacking verifiable grounding in patient-specific evidence. Such characteristics raise substantial medico-legal concerns, as responsibility for erroneous recommendations remains diffuse and current regulatory frameworks do not support autonomous deployment. Accordingly, LLMs are best understood as augmentation tools—supporting clinicians in information retrieval, documentation, and communication—rather than as substitutes for physician judgment, accountability, or longitudinal decision-making. This conclusion aligns with the broader pattern observed in imaging-based AI systems, where high technical performance alone is insufficient to justify clinical autonomy.

### 7.7. Patient-Centered Clinical Outcomes When AI Is Used

Across multiple domains, AI integration has shown the most consistent benefits in improving diagnostic accuracy and workflow efficiency. In breast cancer screening, large-scale real-world data demonstrated that AI-assisted mammography reading increased cancer detection by 17.6% without elevating recall rates, thereby improving diagnostic yield and patient access to early treatment [118]. Similar advances have been reported in colonoscopy, where AI-assisted systems significantly reduce adenoma miss rates and enhance detection of early neoplasia [119,120]. In emergency imaging, triage algorithms for stroke, pulmonary embolism, and pneumothorax have shortened time-to-notification and expedited radiologist review, leading to measurable reductions in door-to-treatment intervals [121,122].

With respect to morbidity and mortality, outcome-level data remain limited, though earlier diagnosis and faster intervention suggest indirect benefits. Preliminary evidence in stroke imaging shows that AI-based systems for large-vessel occlusion detection correlates with faster recanalization times and potential functional improvement, while long-term mortality benefits are yet to be firmly established [3]. Likewise, in population-based mammography, ongoing follow-up studies are assessing interval cancer reduction and survival outcomes following AI deployment.

Conversely, earlier AI systems such as IBM Watson for Oncology underscore the risks of premature implementation. Independent evaluations reported inconsistent and sometimes unsafe treatment recommendations, highlighting the importance of rigorous validation and clinician oversight prior to clinical use. Similarly, commercially deployed sepsis prediction models failed external validation and did not improve patient outcomes, emphasizing that clinical integration must prioritize transparency and reproducibility [123].

From a patient-experience perspective, surveys indicate that individuals generally support AI as a second reader or assistive tool when final decisions remain under physician control [124,125]. Autonomous systems, such as the FDA-cleared IDx-DR for diabetic retinopathy screening, have also improved screening rates in primary care, suggesting that AI can enhance access and satisfaction when applied within appropriate regulatory frameworks [126].

Collectively, these findings highlight that AI most reliably improves diagnostic accuracy, workflow efficiency, and care accessibility, while effects on morbidity, mortality, and patient satisfaction remain promising but incompletely characterized. We have revised the Discussion to include this perspective and appropriately cited recent clinical evidence to provide a balanced, empirically grounded overview of AI’s patient-centered outcomes.

### 7.8. Technological and Implementation Challenges

It should be remembered that AI systems are not ready to “plug and play,” and thus not ready to act autonomously in care settings. Difficulties include system interoperability, integration with hospital information systems (often legacy), the lack of interface standardization, and limited AI–physician interaction within daily clinical work. The time required to commission a system and to implement the insights generated by its use is often so long that the system loses practical value. Radiology is the clearest example: despite the large number of applications developed, only a subset has reached practical, clinician useful deployment [127].

Histopathologic diagnostics, which—like radiology—relies on image-based patterns, likewise faces practical implementation challenges that are not purely technological. Generalizing system performance is typically difficult not only because of possible differences in specimen characteristics but also because of the methods and quality of digitization [128,129,130].

In oncology, a key problem is that AI system outputs reflect the character of data that usually originate from a single institution, which markedly reduces robustness when operating on data from other centers. A system that does not perform with sufficient effectiveness outside the center where it was developed (trained) has little chance of broader implementation success [131,132,133,134].

In cardiology, especially cardiovascular care, barriers include interoperability, costs, and data bias. Model transparency is also problematic: systems must be understandable to end users—clinicians and patients. A lack of understanding of the system’s foundations and of changes required for implementation hinders adoption [135].

Similar observations apply to ECG (electrocardiogram) algorithms, where clinical acceptance is limited by technical integration problems. Software incompatibility prevents rapid or low cost implementation. Inefficient technical rollout and uncertainty about the economic rationale impede uptake and thereby undermine the case for deployment [136].

For clinical decision support systems (CDSSs), some of the most frequent problems are technological hurdles when deploying them into centers with incompatible IT infrastructures. There is also visible difficulty adapting the system to clinicians’ current needs and habits. Systems are built in selected institutions, and subsequent tailoring to the needs of other clinical groups is not always feasible. In discussions of AI adoption risks, financial barriers must not be overlooked (temporary system costs and an unclear time horizon for return on investment after implementation) [137]. When assessing implementation risks, the experience with IBM’s Watson is particularly instructive. Analyses of its deployment reveal a wide range of potential pitfalls, even in settings with substantial financial resources and a well-defined overarching concept. The system’s failure was largely attributed to inadequate clinical validation, limited training data derived primarily from guidelines of a single institution (MSKCC), difficulties in adapting to local clinical practice, and a lack of physician trust due to algorithmic opacity—challenges that are frequently encountered in real-world implementations.

At the same time, it is important to note that certain sepsis prediction models developed using similar data-driven approaches have been associated with approximately an 18% reduction in mortality, underscoring that successful outcomes are possible when validation and integration are carefully addressed [138].

Despite solid technological implementation, insufficient interaction with physicians meant that Watson generated recommendations that diverged from clinical practice, often outdated, which proved unsafe for patients and—despite enormous expenditures—led to the system’s withdrawal [139].

Taken together, these technological and implementation challenges demonstrate that successful clinical deployment of AI depends less on algorithmic performance than on interoperability, workflow integration, economic sustainability, and clinician engagement. Without careful alignment with existing clinical infrastructures and practices, even technically robust AI systems are unlikely to achieve sustained adoption or deliver meaningful clinical benefit.

## 8. Conclusions

AI systems are highly promising as a future technological trend in medicine. In many fields (e.g., radiology and histopathology), they have achieved practical use owing to analytic shape detection capabilities that are more reproducible and precise than those of humans. Nevertheless, when considering broader-scale adoption of AI in medicine, one must keep in mind the existing implementation barriers, the most important of which are communication and integration barriers as well as economic factors (uncertain cost effectiveness of expensive systems). These technological and organizational barriers also have direct financial consequences. Limited interoperability, heterogeneous data standards, and inefficient physician–AI integration frequently result in prolonged implementation timelines and costly customization efforts. When such systems fail to integrate smoothly into clinical workflows, hospitals may incur sunk costs—investments in infrastructure, software, and training that yield limited operational benefit. This reduces utilization efficiency and delays the expected return on investment, thereby increasing institutional hesitation to deploy or expand AI solutions. As discussed in Section 6, these economic uncertainties reinforce the broader conclusion that the near-term trajectory of AI in medicine depends not only on technical readiness and regulatory compliance but also on demonstrable cost-effectiveness and financial sustainability. Optimal integration of AI into clinical workflows requires a human-centered and ethically aligned design [140]. AI systems should function as assistive diagnostic tools embedded within existing PACS or reporting environments, providing interpretable outputs, uncertainty estimates, and visual explanations that support—rather than supplant—clinician judgment. Mandatory human-in-the-loop verification ensures that final responsibility for interpretation and patient management remains with the physician [141]. To mitigate automation bias, clinical protocols should include alternating or blinded review strategies, documentation of AI influence on decision-making, and targeted training in cognitive bias awareness [142]. Furthermore, transparent informed consent processes should clarify that AI serves as an adjunct to expert review, while communication of results and counseling must remain the clinician’s responsibility [143]. Such workflow engineering preserves the patient–physician relationship, enhances patient trust, and aligns AI deployment with core medical ethics.

### 8.1. Implications Supporting the Clinical Use of AI

The potential of artificial intelligence (AI) in medicine is supported by several well-documented advantages, including higher precision and reproducibility in medical imaging, improved standardization and objectification of assessments, and the ability to discover hidden signals and associations beyond human-defined labels. Further strengths include the knowledge and communication competencies of large language models, which can support medical education and provide patient-facing explanations that are often perceived as more detailed or empathetic, as well as the capacity of self-supervised pretraining to close information gaps in data-scarce clinical settings. In practical applications, AI systems have demonstrated value in automating well-defined tasks, assisting clinical workflows, and enhancing decision support, contributing to improved efficiency and consistency in selected diagnostic and organizational processes.

### 8.2. Constraints Limiting Full Physician Replacement

Conversely, substantial limitations constrain the clinical impact of AI and argue against physician replacement. These include unresolved issues of legal liability, the rigidity of algorithmic systems compared with human cognitive flexibility, persistent out-of-distribution limitations in imaging, and the superior ability of clinicians to manage atypical or rare cases. Additional barriers arise from the absence of physical examination and sensory data, the embodiment and manual skill gap, and known limitations of large language models, including hallucinations and overconfidence. From an implementation perspective, AI models often perform well only on data similar to their training distributions, with efficacy declining across different populations, diseases, or care settings; although notable successes exist, such as sepsis prediction systems associated with mortality reductions of approximately 18%, large-scale adoption remains limited, with only a minority of healthcare organizations achieving sustained deployment. Reports indicate that many AI projects fail to deliver a positive return on investment, remain in prolonged pilot phases, and operate within an environment characterized by hype-driven expectations, unclear validation standards, and persistent governance challenges. As a result, AI in medicine has not replaced healthcare professionals but functions primarily as an assistive technology, highlighting the need for future approaches that integrate improved generalization, robust governance, and cognitively grounded models inspired by human reasoning.

### 8.3. Future Directions

Future research should focus on several priorities essential for responsible clinical integration of artificial intelligence. First, standardized evaluation frameworks are needed to assess AI systems beyond isolated performance metrics, incorporating robustness, uncertainty estimation, out-of-distribution behavior, and human–AI interaction effects. Second, longitudinal clinical impact studies are required to determine how sustained AI use influences patient outcomes, clinician behavior, diagnostic vigilance, and potential deskilling over time. Third, closer alignment between technical development, clinical validation, and regulatory frameworks is necessary to ensure transparency, accountability, and harmonized standards across jurisdictions. Addressing these priorities will be critical to moving AI in medicine from task-level performance demonstrations toward safe, evidence-based, and ethically grounded clinical augmentation.

Based on the current evidence, artificial intelligence will not replace physicians in the foreseeable future but will increasingly augment clinical practice by automating well-defined tasks while leaving integrative judgment, physical examination, procedural skills, and accountability firmly in human hands.

## Figures and Tables

**Table 1:** Conceptual framework of physician functions according to their degree of automability.

Category	Physician Function Type	Representative Examples	Current AI Capability	Principal Limitations
Largely replaceable (task-level automation)	Highly standardized, data-driven analytic tasks	Image triage, lesion detection, quantitative measurements, structured reporting, documentation drafting	Near-human or superhuman performance in narrowly defined tasks	Limited generalization beyond training data; vulnerability to out-of-distribution cases; requirement for human supervision
Augmentable (human-in-the-loop)	Context-dependent cognitive and interpretive tasks	Diagnostic reasoning support, risk stratification, differential diagnosis assistance, treatment planning support	Decision support and recommendation generation	Lack of embodied cognition; limited uncertainty awareness; dependence on clinician judgment and accountability
Not automatable (structural human functions)	Embodied, ethical, and responsibility-bearing clinical tasks	Physical examination, palpation, procedural and surgical skills, empathic communication, informed consent, final clinical responsibility	Not achievable with current AI systems	Absence of sensorimotor embodiment, multisensory integration, moral agency, and legal responsibility

**Table 4:** Representative studies illustrating the performance and limitations of artificial intelligence in selected clinical tasks.

Clinical Task	Modality	AI Model Type	Representative Study (Ref.)	Reported Performance (Typical)	Key Limitation(s)
Intracranial hemorrhage detection	CT	Convolutional neural network	Titano et al., 2018 [2]	Sensitivity ≈ 0.90–1.00; AUC > 0.90	Limited generalization across scanners and institutions; false positives require radiologist oversight
Pulmonary embolism detection	CT pulmonary angiography	CNN-based triage systems	Ardila et al., 2019 [8]	AUC ≈ 0.90–0.94; sensitivity up to ~0.95	Not validated for autonomous diagnosis; performance sensitive to data distribution
Breast cancer detection	Mammography	Deep learning CAD systems	Rodríguez-Ruiz et al., 2019 [3]; Salim et al., 2020 [4]	AUC ≈ 0.94–0.97; comparable to expert radiologists	Screening-only context; limited applicability to symptomatic patients
Pneumonia detection	Chest X-ray	CNN architectures	Zech et al., 2018 [30]	AUC > 0.90 (internal validation)	Marked performance degradation on external datasets (domain shift)
MRI image reconstruction	MRI	Deep learning reconstruction networks	Knoll et al., 2020; fastMRI [96]	SSIM often > 0.95	Risk of hallucinated or missing anatomical structures
Medical knowledge assessment	Text (MCQs)	Large language models	Kung et al., 2023 [23]	Accuracy > 0.85 on USMLE-style exams	Reflects pattern recognition; not equivalent to real-world clinical reasoning
Patient-facing written communication	Text	Large language models	Ayers et al., 2023 [22]	Responses rated as detailed and empathetic	Susceptibility to hallucinations; lack of accountability

## Data Availability

No new data were created or analyzed in this study. Data sharing is not applicable to this article.

## Abbreviations

The following abbreviations are used in this manuscript:

ACC	Accuracy
AI	Artificial Intelligence
AUC	Area Under the Curve (ROC)
AUROC	Area Under the Receiver Operating Characteristic Curve
BACC	Balanced Accuracy
BERT	Bidirectional Encoder Representations from Transformers
CDSS	Clinical Decision Support System(s)
CE (marking)	Conformité Européenne
CNN/CNNs	Convolutional Neural Network(s)
CT	Computed Tomography
ECG	Electrocardiogram
EU	European Union
F1Score/F1	Harmonic Mean of Precision and Recall
GPT	Generative Pretrained Transformer
HbA1c	Glycated Hemoglobin A1c
IT	Information Technology
LLM/LLMs	Large Language Model(s)
MAE	Mean Absolute Error
MSKCC	Memorial Sloan Kettering Cancer Center
MRI/MR	Magnetic Resonance Imaging/Magnetic Resonance
OOD	Out of Distribution
PACS	Picture Archiving and Communication Systems
PET	Positron Emission Tomography
ROI	Return on Investments
RIS	Radiology Information System
TCAV	Testing with Concept Activation Vectors
USMLE	United States Medical Licensing Examination
WER	Word Error Rate
XAI	Explainable AI
